# Genome-scale portrait and evolutionary significance of human-specific core promoter tri- and tetranucleotide short tandem repeats

**DOI:** 10.1186/s40246-018-0149-3

**Published:** 2018-04-05

**Authors:** N. Nazaripanah, F. Adelirad, A. Delbari, R. Sahaf, T. Abbasi-Asl, M. Ohadi

**Affiliations:** 1 Iranian Research Center on Aging, University of Social Welfare and Rehabilitation Sciences, Tehran, Iran; 2 Department of Biostatistics, University of Social Welfare and Rehabilitation Sciences, Tehran, Iran

**Keywords:** Short tandem repeat, Core promoter, Human-specific, Trinucleotide, Tetranucleotide

## Abstract

**Background:**

While there is an ongoing trend to identify single nucleotide substitutions (SNSs) that are linked to inter/intra-species differences and disease phenotypes, short tandem repeats (STRs)/microsatellites may be of equal (if not more) importance in the above processes. Genes that contain STRs in their promoters have higher expression divergence compared to genes with fixed or no STRs in the gene promoters. In line with the above, recent reports indicate a role of repetitive sequences in the rise of young transcription start sites (TSSs) in human evolution.

**Results:**

Following a comparative genomics study of all human protein-coding genes annotated in the GeneCards database, here we provide a genome-scale portrait of human-specific short- and medium-size (≥ 3-repeats) tri- and tetranucleotide STRs and STR motifs in the critical core promoter region between − 120 and + 1 to the TSS and evidence of skewing of this compartment in reference to the STRs that are not human-specific (Levene’s test *p* < 0.001). Twenty-five percent and 26% enrichment of human-specific transcripts was detected in the tri and tetra human-specific compartments (mid-*p* < 0.00002 and mid-*p* < 0.002, respectively).

**Conclusion:**

Our findings provide the first evidence of genome-scale skewing of STRs at a specific region of the human genome and a link between a number of these STRs and TSS selection/transcript specificity. The STRs and genes listed here may have a role in the evolution and development of characteristics and phenotypes that are unique to the human species.

## Introduction

Speciation and evolution are, at least in part, due to the plasticity (expansion or contraction) of short tandem repeats (STRs)/microsatellites, which can function as “tuning knobs” in response to the environment or other genes [1–3]. In line with the above, certain STRs are directionally expanded in the human species or co-occur identically in related taxa such as primates [4–8]. Genes that contain STRs in their promoters have higher expression divergence compared to genes with fixed or no STRs in the gene promoters [9]. Recent reports indicate a role of repetitive sequences in the rise of young transcription start sites (TSSs) in human evolution [10–12].

Preliminary data on the sequencing of a number of “exceptionally long” STRs (≥ 6-repeats), which compose 1–2% of all human core promoter STRs [3], support critical evolutionary adaptive roles for a number of these STRs. Human specificity of the predominant allele of the *RIT2* core promoter STR in the human species, the presence of the shortest allele of this STR (5-repeat) in hunter-gatherer humans (BUSHMAN KB1: rs113265205), the lack of this allele in the agricultural modern humans (Genome Aggregation database: gnomad.broadinstitute.org), and its co-occurrence with schizophrenia provide the first indication of STR allele selection in humans [13]. A link between the *CYTH4* core promoter STR (the longest tetranucleotide STR identified in a human gene core promoter) with the Old World monkeys and Apes and evidence of extreme “disease-only” genotypes at this STR with schizophrenia [14] provide the first link between a primate-specific STR and higher-order brain functions in human. The “exceptionally long” CA-repeat in the core promoter of *SCGB2B2* is another example of directional STR expansion in the Old World monkeys and Apes [5]. The *PAXBP1* gene is an extreme example in which expansion of a core promoter CT-repeat occurs in the Old World monkeys and reaches maximum length and complexity in human; OMIM: 617621 [4].

As “exceptionally long” STRs may be subject to natural selection, short- and medium-size alleles (≥ 3-repeats) might have had similar fate. This is indicated by the predominance of specific short- and medium-size penta- and hexanucleotide STRs and their cognate transcription factors (TFs) in the critical core promoter interval [15]. Indeed, shortening of a number of STRs and their identical co-occurrence is linked to the evolution of primates [8]. In line with the above findings, repeats associated with younger human TSSs tend to be shorter than those in older TSSs [10]. In the study reported here, we present genome-scale data on two categories of STRs, i.e., tri- and tetranucleotide STRs, and their implication in human evolution.

## Materials and methods

The interval between − 120 and + 1 to the TSS of all human protein-coding genes annotated in the GeneCards database (version 3.0) (www.genecards.org) was screened for tri- and tetranucleotide STRs of ≥ 3-repeats, based on the Ensembl database (versions 87-91) (asia.ensembl.org) and using the Microsatellite Repeats Finder at the following link: http://insilico.ehu.es/mini_tools/microsatellites/

The evolutionary status of the identified STRs was analyzed in 25 species (*N*), including primates (*N* = 5), non-primate mammals (*N* = 12), birds and reptiles (*N* = 5), amphibians (*N* = 1), and fish (*N* = 2), based on the Ensembl database.

Human specificity of transcripts was evaluated based on the multiple and pair-wise %identity scoring of the TSS-flanking 5′ untranslated region (UTR), using the sequence alignment program Clustal Omega (https://www.ebi.ac.uk/Tools/msa/clustalo), and the overall composition of the transcript and encoded protein (i.e., length of the transcript, number of exons and amino acids). The threshold of sequence identity was set at 50%, which was based on the comparison of two randomly selected and unrelated sequences in the human genome.

The *p* value for the skewing of the human-specific STR compartment was calculated using Levene’s equality of variances test.

The *p* values for transcript enrichment were calculated using the two by two table analysis;

the human-specific tri- and tetranucleotide STR groups were compared against corresponding randomly selected STRs from the non-human-specific STRs. The comparison was set based on the sample size of the human-specific STRs (*n*) and the sample size of the non-human compartments (1.5n).

## Results

### Overall prevalence of tri- and tetranucleotide STR motifs across human protein-coding core promoter sequences

**Fig. 1:**
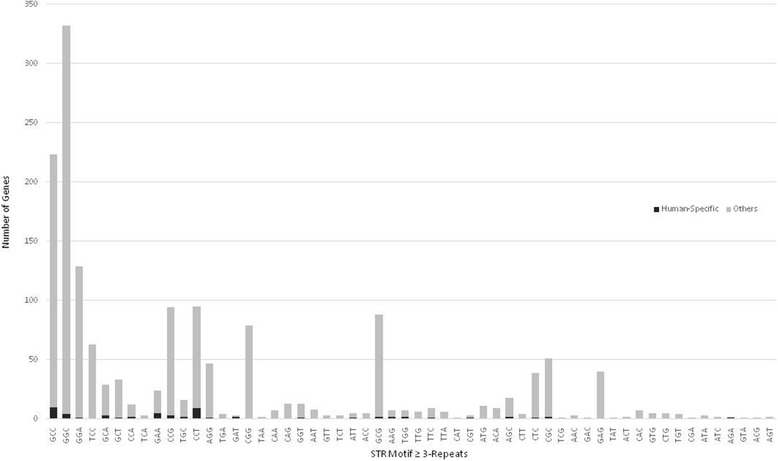
Genome-scale prevalence of human protein-coding core promoter trinucleotide STRs and significant skewing of the human-specific STR compartment

**Fig. 2:**
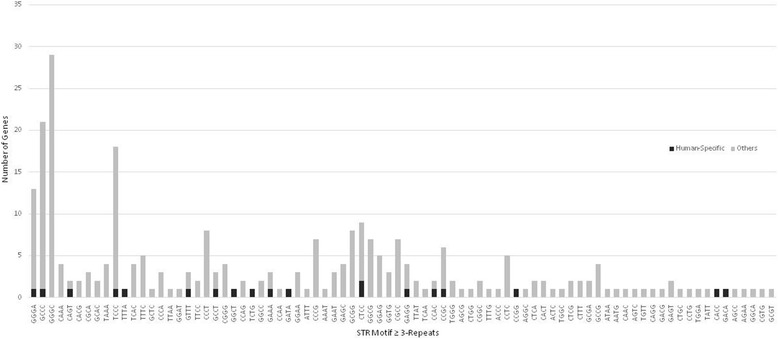
Genome-scale prevalence of human protein-coding core promoter tetranucleotide STRs and significant skewing of the human-specific STR compartment

In total, 56 and 82 STR motifs were detected for the tri- and tetranucleotide repeats, respectively (Figs. 1 and 2). The most prevalent tri- and tetranucleotide STR motifs across the human protein-coding gene core promoters were GGC and GGGC, respectively (Figs. 1 and 2). In the category of non-GC STRs, GGA and TCCC were the most prevalent tri- and tetranucleotides, respectively.

### Skewing of the human-specific core promoter tri- and tetranucleotide STRs

A significant skewing of the tri- and tetranucleotide STR distribution was found in the human-specific tri- (Fig. 1) and tetranucleotide (Fig. 2) compartments (Levene’s *p* < 0.001). While the most prevalent tri- and tetranucleotide repeats in the non-human-specific category were the GGC- and GGGC-repeats, respectively, the most prevalent human-specific STRs were of the GCC and CTCC motifs, respectively. Disproportionate distribution of human-specific STRs was also detected in other STRs such as CCT, GAA, CTCC, GTTT, and GAAA.

The human-specific tri- and tetranucleotide STRs were of a wide range of motifs, e.g., the CCA motif in *ADCY6*, the TCCC motif in *ARHGEF35*, GCCC in *DRD2*, and GTTT in *MCTP2* (Tables 1 and 2)*.*

**Table 1:** Genome-scale human-specific core promoter trinucleotide STRs

Human gene symbol	Ensembl transcript ID	Variant no.	STR formula	
*ADCY6*	ENST00000307885.4	201	− 48 (CCA)3	
*AMY1C*	ENST00000370079.3	201	− 79 (ATT)3	
*APBB1*	ENST00000311051.7	202	− 11 (GCC)3	
*BRINP2* (*FAM5B*)	ENST00000361539.4	201	− 26 (GGC)5	
*BVES*	ENST00000446408.2	203	− 35 (CCT)3	
*CCDC178* (*C18orf34*)	ENST00000300227.12	201	− 79 (AGC)3	− 57 (CGC)5
*CDH4*	ENST00000543233.2	201	− 31 (CCT)3	
*CIAPIN1*	ENST00000563341.1	202	− 39 (CCT)3	
*CNTNAP2*	ENST00000361727.7	201	− 98 (TGC)3	
*CST4*	ENST00000217423.3	201	− 83 (GGA)3	
*CYP4A11*	ENST00000310638.8	201	− 86 (CCT)3	
*C1orf204*	ENST00000368102.5	201	− 11 (AAG)3	
*C22orf24*	NM_015372.2.1		− 98 (GCA)3	
*C3AR1*	ENST00000546241.1	202	− 70 (AGA)3	
*DDX58*	ENST00000379868.5	201	− 33 (CCT)3	
*ECSCR*	NM_001077693.3.1		− 121 (CCA)3	
*GRIN2D*	ENST00000263269.3	201	− 67 (GCC)3	
*GSDMB*	ENST00000394175.6	203	− 55 (GGC)3	
*INPP4B*	ENST00000503927.5	202	− 88 (CGC)3	
*KBTBD12*	ENST00000407609.7	204	− 57 (CCT)3	
*KIAA1211*	ENST00000504228.5	203	− 81 (AAG)3	
*KRAS*	ENST00000311936.7	202	− 89 (GAA)3	
*KTN1*	ENST00000395308.5	202	− 70 (GCG)9	
*LACTBL1*	ENST00000426928.6	201	− 58 (GAA)3	
*LCE2B*	ENST00000368780.3	201	− 79 (CCT)3	
*LCOR* (*C10orf12*)	ENST00000356016.7	202	− 97 (CCT)3	− 108 (GCC)3
*MPRIP*	ENST00000466186.2	209	− 88 (GCA)11	
*MSANTD3* (*C9orf30*)	ENST00000374885.5	201	− 25 (GCC)7	
*NPAS1*	ENST00000439365.6	201	− 135 (GAA)9	
*OR4X1*	ENST00000320048.1	201	− 100 (GAT)3	
*PABPC1L2B*	ENST00000373521.3	201	− 60 (GCC)3	
*PAQR9*	ENST00000498470.1	203	− 29 (TGC)3	
*PRSS1*	ENST00000492062.1	205	− 96 (GAT)3	
*RGPD6*	ENST00000455695.1	205	− 70 (GGC)5	
*RNF215*	ENST00000215798.10	201	− 123 (GCT)5	
*R3HDM2*	ENST00000448732.1	208	− 27 (GCC)3	
*SCN3B*	ENST00000299333.7	201	− 29 (GGT)3	
*SERPINB9*	ENST00000380698.4	201	− 12 (GCA)3	
*SIGLEC7*	ENST00000305628.7	201	− 80 (TTC)3	
*SPATC1L* (*C21orf56*)	ENST00000330205.10	202	− 37 (TGG)4	−90 (TGG)4
*STUB1*	ENST00000219548.8	201	− 91 (GCC)3	
*SUMF1*	ENST00000272902.9	201	− 102 (AGC)3	
*TEX12*	ENST00000280358.4	201	− 105 (TGG)3	
*TMEM99*	ENST00000301665.7	201	− 32 (CCG)3	− 47 (CCG)3
			− 59 (CCG)3	− 83 (CCG)3
			− 110 (CCG)3	− 125 (CCG)3
			− 48 (GCC)3	− 60 (GCC)3
			− 84 (GCC)3	− 126 (GCC)3
*TNNC2*	ENST00000372557.1	202	− 53 (GCC)3	
*TPTE*	ENST00000427445.6	201	− 110 (GCG)3	
*TRBJ2-7*	ENST00000390419.1	201	− 120 (GGC)3	
*TRGV5*	NC_000007.14:TRGV5:u_t_1.1		− 48 (CTC)3	
*TRIM39*	ENST00000376656.8	201	− 58 (CCT)4	
*UAP1*	ENST00000367926.8	204	− 9 (CGT)3	
*VNN2*	ENST00000326499.10	201	− 31 (GAA)10	
*WRN*	NM_000553.4.1		− 67 (GCC)3 − 69 (CCG)4	− 92 (GCC)3
*WRNIP1*	ENST00000618555.4	205	− 67 (CCG)3	
*ZDHHC21*	XM_006716760.1.1		− 32 (AGG)3	
*ZSCAN30*	ENST00000639929.1	212	− 57 (GAA)3	

The numbers before the brackets represent the start site of the STR in respect of the corresponding transcription start site. “Variant no” corresponds to the Ensembl isoform number

**Table 2:** Genome-scale human-specific core promoter tetranucleotide STRs

Human gene symbol	Ensembl transcript ID	Variant no.	STR formula
*ARHGAP5*	ENST00000345122.7	202	− 110 (GGGA)4
*ARHGEF35*	ENST00000378115.2	201	− 22 (TCCC)3
*ARL17B*	ENST00000622877.4	201	− 99 (CTCC)3
*ATP7A*	ENST00000343533.9	201	− 27 (GAGG)3
*DRD2*	ENST00000542616.1	207	− 54 (GCCC)3
*DUX4*	ENST00000565211.1	203	− 144 (GGCT)6
*FAM83G*	ENST00000388995.10	202	− 80 (TCTG)3
*GTF2IRD2B*	ENST00000614064.4	206	− 17 (GAAA)3
*JCAD* (*KIAA1462*)	ENST00000375377.1	201	− 13 (CCGG)3
*MCTP2*	ENST00000451018.7	203	− 102 (GTTT)3
*METTL21C* (*C13orf39*)	ENST00000267273.6	201	− 69 (CAGT)3
*OR10G6*	ENST00000307002.3	201	− 123 (GATA)13
*PHYHD1*	ENST00000308941.9	201	− 107 (TTTA)3
*SAMD1*	ENST00000269724.5	201	− 75 (CCGC)3
*TEAD4*	ENST00000540314.1	206	− 51 (CTCC)3
*TRAJ49*	ENST00000390488.1	201	− 124 (GCCT)7
*TRDJ2*	ENST00000390475.1	201	− 86 (CCAC)3
*TRAV38-1*	ENST00000390464.2	201	− 109 (CACC)3
*TRAV7*	ENST00000390429.3	201	− 111 (GACA)3

The numbers before the brackets represent the start site of the STR in respect of the corresponding transcription start site. “Variant no” corresponds to the Ensembl isoform number

In a number of instances, not only the STR, but also the genes containing those STRs, were human-specific (e.g., *ARHGEF35*, *AMY1C*, and *C1orf204*). Furthermore, a number of the tri- and tetranucleotide STRs were found to be unique to the human species at the specified interval of − 120 to + 1 TSS. For example, in the tetranucleotide compartment, CACC, GACA, CCGG, GATA, TCTG, GGCT, and TTTA STRs were detected in human only.

### Enrichment of human-specific transcripts at the human-specific STR compartment

**Fig. 3:**
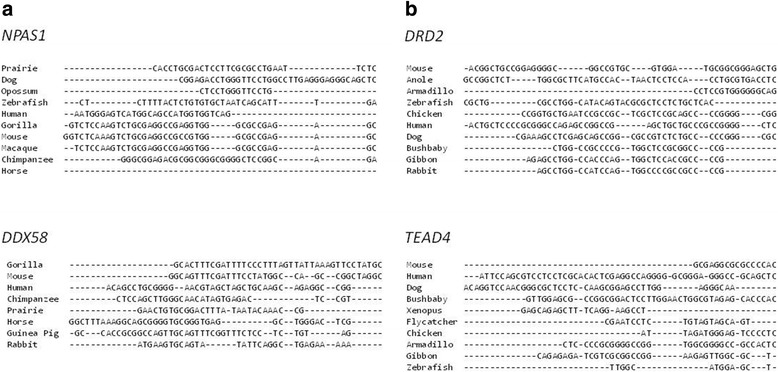
Multiple sequence alignment of the TSS-flanking 5′UTRs. Examples of ClustAl Omega sequence alignment are represented in the tri- (**a**) and tetranucleotide (**b**) categories. Species inclusion was based on the information available in the Ensembl database

**Fig. 4:**
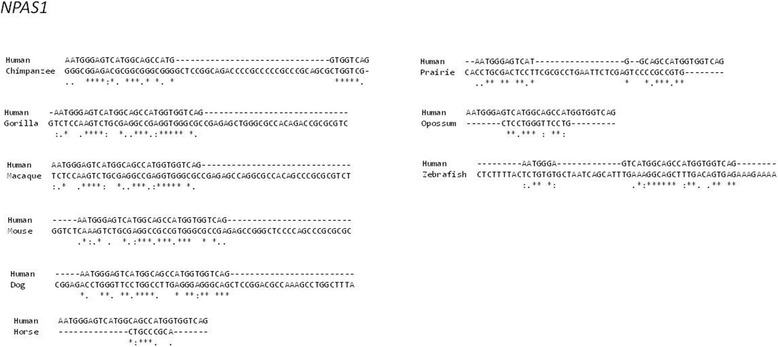
Pair-wise sequence comparison of the TSS-flanking 5′UTRs. %identity scoring was performed between human and other species. Asterisks represent sequence identity

Based on sequence comparison and the overall composition of the transcript and encoded protein, 25 and 26% of the transcripts in the tri and tetra human-specific compartments were found to be human-specific (mid-*p* < 0.00002 and mid-*p* < 0.002), respectively). The %identity score of multiple sequence alignment for the human-specific transcripts was 0 (exemplified in Fig. 3), and pair-wise analysis (exemplified in Fig. 4) resulted in %identity scores ranging from 37 to 48%. In the trinucleotide category, 14 genes, *MPRIP*, *NPAS1*, *PAQR9*, *PRSS1*, *R3HDM2*, *TMEM99*, *ZSCAN30*, *C22orf24*, *ECSCR*, *AMY1C*, *DDX58*, *C1orf204*, *RGPD6*, and *LCE2B*, contained human-specific transcripts. In the tetranucleotide category, five genes, *DRD2*, *DUX4*, *TEAD4*, *ARL17B*, and *ARHGEF35*, contained human-specific transcripts.

A number of the identified STRs were linked to noncanonical translation in the following genes, *TEAD4*, *ECSCR*, *MPRIP*, *PAQR9*, *PRSS1*, and *ZSCAN30.*

## Discussion

There is an ever-growing literature on the biological and pathological implications of STRs at the inter- and intraspecies levels [16–27]. The STRs listed in the present study are genetic codes that are unique to humans and are likely to be responsible for the human-specific regulation of the relevant genes. The significant enrichment of human-specific transcripts at the human-specific STR compartment indicates a link to a mechanism for TSS selection and transcript specificity.

A number of the identified STRs such as GTTT have established repressor activity [6, 28, 29] and are differentially expanded in certain genes in the Old World monkeys and Apes [14]. Purine STRs such as GAAA repeats are also functional in gene expression regulation, and their link to certain diseases unique to humans were previously reported [30, 31]. While the CG-rich STRs (e.g., CCG, GGGC) are subject to DNA methylation and can repress gene expression activity [32], they can also form G4 quadruplex structures, which have significant functions in gene expression regulation [33]. Several other identified STRs can form G4 structures with high overlap fraction (e.g., AGGG/CCCT, GCCC/GGGC).

It is not possible to estimate the number of crucial events that have led to the emergence of the human species. However, only a few genetic changes are needed to spur the evolution of new species in general, exemplified by the highly restricted initial divergence in butterfly hybridization models [34]. Accelerated evolution of a number of the identified genes in the present study (e.g., *DRD2*) has a well-established role in the origin of *Homo sapiens* [35]. Remarkably, a human-specific 7-amino acid transcript of this gene is flanked by a human-specific GCCC-repeat. Human-specific transcripts are increasingly recognized of having a role in the pathogenesis of diseases unique to the human species, such as schizophrenia [25, 36].

In a number of instances, not only the STR and the transcript, but also the gene containing these STRs and transcripts, were unique to humans, e.g., *AMY1C*, which is indicated in the evolution of the human phenotype during the Pleistocene [37].

For a number of the identified genes, sparse literature is available on the relevant function and pathways (e.g., *ARHGEF35*, *CXorf40A*, *C22orf24*, *TMEM99*, and *ARL17B*).

In a number of the identified genes, the STRs were linked to noncanonical (non-AUG) translation. Although the significance of this compartment is unknown for the most part, recent emerging data indicates likely biological functions [38].

The plasticity of STRs confers them unique ability to respond to adaptive evolutionary processes in a more efficient way than the quaternary codes provided by the SNSs. This potential aspect of STRs is vastly unknown at present, and it is expected that identification of STRs that have evolved differentially in humans vs. other species may pave the way for better understanding of the evolutionary implication of these highly mutable motifs.

This study warrants expansion to other vitally important gene regulatory sequences such as the distal promoter, 5′UTR, and 3′UTR. It is also necessary to sequence these STRs in characteristics and diseases that are unique to the human species. The recent reports of mass STR analysis using CRISPR/Cas9 [39] make it particularly more feasible to investigate STRs in the context of human evolution.

## Conclusion

Our findings provide the first evidence of genome-scale skewing of STRs at a specific region of the human genome, and support a link between STRs and TSS selection/transcript specificity. The genes and STRs listed here may have a role in the divergence of humans from other species through the development of characteristics and phenotypes that are unique to the human species.

## Abbreviations

SNS: Single nucleotide substitution
STR: Short tandem repeat
TF: Transcription factor
TSS: Transcription start site
UTR: Untranslated region
